# Food Adulterations

**Published:** 1880-04

**Authors:** 


It is enough to shake our confidence in human nature, to find that there are men, who, for the sake of gain, will bring torture and death, not only to their fellow-beings, but upon meek defenceless animals that patiently wear out their lives in our service. Not long since an English paper announced that in Manchester many horses had died after intense suffering. The cause of death was investigated, and it was found that the dealer who supplied the food with which the horses had been fed, had added a quantity of plaster of paris to give it additional weight, and that this plaster with the food and water formed a solid mass which completely obstructed the passage through the stomach and intestines.

It is well known in our own country that there are manufacturers who sell clay for red peper, alum for cream of tartar, and who deal out destruction to little children in the form of candies made from glucose and kaolin, colored with vermilion. It is high time that something were done to abate, if not abolish these abuses. Legislation seems powerless to do so, and the only way appears to be, to expose these fraudulent practices and instruct the people how to escape them. The counterfeit which is to-day doing the greatest and most wide-spread mischief, is the substitution of alum for cream of tartar. In certain instances, alum, when taken into the stomach has acted as a poison; and many cases of death have occurred from it. Its repeated contact, even in small quantities, acts destructively on mucus membranes, producing a chronic irritation of the throat, stomach and intestinal canal. The most painful and obstinate cases of dyspepsia have been caused by the long continued use of food containing alum. We formerly advised people to procure pure materials and make their own baking powders; but we found from experience that this was impracticable, for the reason that it is difficult to find pure cream ef tartar, and still more difficult to combine the materials in the proper proportions, if we had them pure. We conclude, therefore, that the safest plan, and the most economical, is to purchase baking powders that are made by reliable mannfacturers. There are a few reliable concerns of this kind; but recent investigations indicate that there are ten that adulterate their goods, to one that sells a pure article.

We recently purchased in a neighboring city, for examination, one pound of loose baking powder—that is, not canned—and four cans of one pound each, of different makers, whose goods we knew nothing about. We found the package of loose baking powder composed wholly of crude carbonate of soda, or soda ash, and alum. Can No. 1 was a mixture of bi-carbonate of soda, alum, tartaric acid and corn starch. No. 2 was bi-carbonate of soda, alum and flour. No. 3, bi-carbonate of soda, alum and a trace of tartaric acid. No. 4 was found perfectly pure, and had the ingredients combined in proper proportions. We feel that it is not only our duty, but a pleasure to state that this, the only sample out of five, that was found made of pure grape cream of tartar, and entirely free from alum, ammonia,, and all adulterations, was Cleveland's Superior Baking Powder, manufactured by Cleveland Brothers, Albany, N. Y.

